# Disseminated nocardiosis in a patient with AIDS and B-cell non-Hodgkin’s lymphoma: a case report

**DOI:** 10.1186/s12879-024-10413-0

**Published:** 2025-01-06

**Authors:** Delvis R. Reverón, David M. Flora-Noda, Lily M. Soto, Maribel Dolande, Juan Frey, Aleiram Chaurio, Bárbara D. Ruiz-Alayón, Jocays Caldera, Fhabián S. Carrión-Nessi, David A. Forero-Peña

**Affiliations:** 1 Department of Infectious Diseases, Hospital Universitario de Caracas, Caracas, Venezuela; 2 Department of Mycology, Instituto Nacional de Higiene “Rafael Rangel”, Caracas, Venezuela; 3 Department of Internal Medicine, Hospital Universitario de Caracas, Caracas, Venezuela; 4 “Luis Razetti” School of Medicine, Universidad Central de Venezuela, Caracas, Venezuela; 5 Biomedical Research and Therapeutic Vaccines Institute, Ciudad Bolívar, Venezuela

**Keywords:** *Nocardia*, Nocardiosis, Human immunodeficiency virus, Non-hodgkin’s lymphoma, Case reports, Venezuela

## Abstract

**Background:**

Disseminated nocardiosis is a rare and potentially fatal disease, with a higher incidence in immunocompromised patients, such as those living with human immunodeficiency virus (HIV) or hematological malignancies, including lymphoma. Information on *Nocardia* spp. infection in Venezuela is limited.

**Case presentation:**

We present the case of a 62-year-old male patient, recently diagnosed with HIV, who exhibited prolonged fever and unintentional weight loss. Paraclinical tests revealed pancytopenia and a marked elevation of lactate dehydrogenase. Disseminated histoplasmosis was suspected, prompting a bone marrow (BM) aspirate. Culture and molecular studies for *Histoplasma* spp. and *Mycobacterium tuberculosis* in BM samples were negative. Antiretroviral therapy with tenofovir/lamivudine/dolutegravir was initiated, but the patient subsequently experienced clinical deterioration, including ascites, pericardial effusion, and respiratory failure. Post-mortem biopsy and immunohistochemistry identified non-Hodgkin’s lymphoma of B-cell lineage, and mycological culture of BM isolated *Nocardia farcinica*.

**Conclusion:**

Disseminated nocardiosis may mimic histoplasmosis. *Nocardia* spp. infection should be considered in HIV patients, particularly in advanced stages of infection.

## Background

Nocardiosis is a rare infectious disease caused by gram-positive aerobic bacteria with a filamentous and branched appearance, partially acid-alcohol resistant, belonging to the family Nocardiaceae, within the order Actinomycetales [1]. *Nocardia* spp. infections are acquired through inhalation of the microorganism or direct skin inoculation, presenting clinically as pulmonary or cutaneous disease, and may also disseminate from these initial foci to other organs [2]. Risk factors include deficiencies in cellular immunity, such as those related to human immunodeficiency virus (HIV) infection, solid organ transplantation, or malignant neoplasms [3–5].

Most cases of nocardiosis involve pulmonary manifestations, presenting as pneumonia, endobronchial inflammatory masses, lung abscesses, or cavitary disease [6]. These presentations may mimic a range of pulmonary infections, including pulmonary tuberculosis [7] and *Aspergillus* pneumonia [8]. Additionally, nocardiosis may present as various types of skin and soft tissue infections [9], often resulting in delayed or missed diagnoses. For example, immunocompetent hosts may develop primary cutaneous nocardiosis with lymphogenic spread, mimicking sporotrichosis [10, 11]. Nocardiosis may also resemble brain or lung metastases, occurring in both immunocompromised [12–14] and immunocompetent patients [15, 16]. These diverse and nonspecific presentations have earned *Nocardia* the moniker of a “great imitator”.

Diagnosing *Nocardia* spp. infections remains challenging due to the complexity of microbiological identification in routine laboratories. In Venezuela, most studies on *Nocardia* spp. have focused on mycetoma samples [17, 18], primarily from the central-western regions, including Lara and Falcon states [19]. A 2011 study analyzed 53 strains from patients with actinomycetoma and nocardiosis, identifying several species using molecular methods: *N. brasiliensis*, *N. otitidiscaviarum*, *N. farcinica*, *N. nova*, *N. cerradoensis*, *N. mexicana*, *N. cyriacigeorgica*, and *N. veterana* [20]. More recently, in a case series of 16 patients with mycetoma, *Nocardia* spp. were identified as the etiologic agent in half of the cases [21]. Despite these findings, knowledge of the epidemiology and clinical behavior of nocardiosis in Venezuela remains limited. We present here a rare case of disseminated nocardiosis caused by *N. farcinica* in a patient with HIV and B-cell non-Hodgkin’s lymphoma. This case underscores the diagnostic and therapeutic challenges posed by this opportunistic infection in immunocompromised individuals.

## Case presentation

On January 21, 2024, a male patient without previously known diseases, but with a smoking index > 41, presented with a 3-week history of asthenia, fever (38.5 ºC) preceded by chills with intermittent pattern, and dyspnea on moderate exertion. A review of systems revealed that since January 2023, he had experienced progressive and involuntary weight loss (approximately 20 kg), asthenia, and nocturnal diaphoresis. Additionally, he had 2 months of abdominal distension and diarrhea (without mucus or blood). A fourth-generation HIV enzyme-linked immunosorbent assay test was performed and returned positive. Physical examination documented oropharyngeal candidiasis, and he was prescribed fluconazole 150 mg orally once daily. As prophylaxis for opportunistic infections was indicated TMP-SMX 160/800 mg orally once daily. Additional paraclinical tests were requested. On January 25, 2024, pancytopenia and markedly elevated lactate dehydrogenase (LDH) were documented, leading to his admission to the hospital with a diagnosis of acquired immunodeficiency syndrome (AIDS) and probable disseminated histoplasmosis-type granulomatous disease.

The initial blood analysis was as follows: white blood cells: 2.8 × 10^3^/µL, hemoglobin: 10.1 g/dL, platelets: 135 × 10^3^/µL; creatinine: 0.8 mg/dL, serum sodium: 131 mmol/L, serum potassium: 4.2 mmol/L, corrected calcium: 9.5 mg/dL, erythrocyte sedimentation rate: 80 mm/h; C-reactive protein level: <0.6 mg/dL; LDH: 1,860 U/L; total bilirubin: 0.4 mg/dL; alanine aminotransferase: 44 U/L, aspartate aminotransferase: 113 U/L, alkaline phosphatase: 88 U/L; albumin: 3.4 g/dL. The tuberculin test was negative. T-lymphocyte subpopulation analysis showed CD4^+^ at 44 cells/mm^3^ (10%), CD8^+^ at 345 cells/mm^3^ (78%), and a CD4^+^/CD8^+^ ratio of 0.128. HIV viral load testing was not performed.

**Fig. 1:**
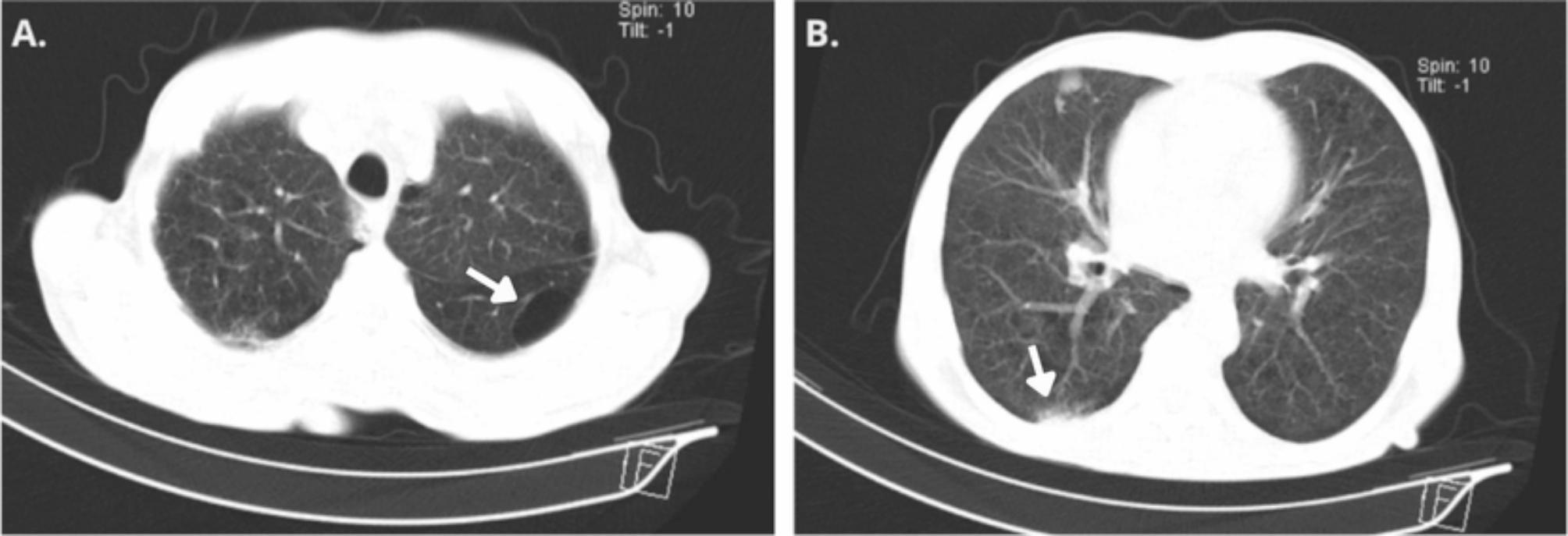
High-resolution chest computed tomography scan. **(A)** Subpleural bulla measuring 5 × 2.5 cm in the apical segment of the left lower lobe. **(B)** Diffuse hypodensity of the lung parenchyma, with hypodense wall images predominantly in the lower lobes

A chest computed tomography scan revealed a subpleural bulla in the apical segment of the left lower lobe (Fig. 1A), diffuse hypodensity of the lung parenchyma, hypodense wall images predominantly in the lower lobes, along with pleural thickening of nodular appearance in the right lung. Confluent lymphadenopathies were also observed in the left pre-vascular chain, without any signs of cavitation or a tree-in-bud pattern (Fig. 1B). A urinary antigen test for *Histoplasma*, performed on January 30, 2024, was negative. On February 1, 2024, antiretroviral therapy (ART) with tenofovir/lamivudine/dolutegravir was initiated. Given the clinical context of pancytopenia, elevated LDH, and persistent fever, lymphoma and other opportunistic infections were ruled out. Due to the absence of palpable adenopathies suitable for biopsy, a bone marrow (BM) aspirate was performed on February 5, 2024, for immunohistochemical analysis and complementary microbiological testing. Polymerase chain reaction for *Histoplasma* and mycobacteria on the BM sample was negative. During hospitalization, the patient developed hypoxemia on February 6, 2024, prompting the initiation of supplemental oxygen and an adjustment of the TMP-SMX regimen to a therapeutic dose (160/800 mg IV every 8 h) due to suspected *Pneumocystis jirovecii* pneumonia. The patient showed a favorable response with a reduction in fever episodes and improvement in hypoxemia, leading to a transition to oral TMP-SMX at the same dose. As the infectious disease team suspected histoplasmosis, they recommended starting treatment. After discussion with the patient and family, itraconazole was initiated with a loading dose, followed by 200 mg once daily for 6 to 12 weeks. The patient was discharged on February 9, 2024, with instructions to continue taking TMP-SMX for 21 days.

**Fig. 2:**
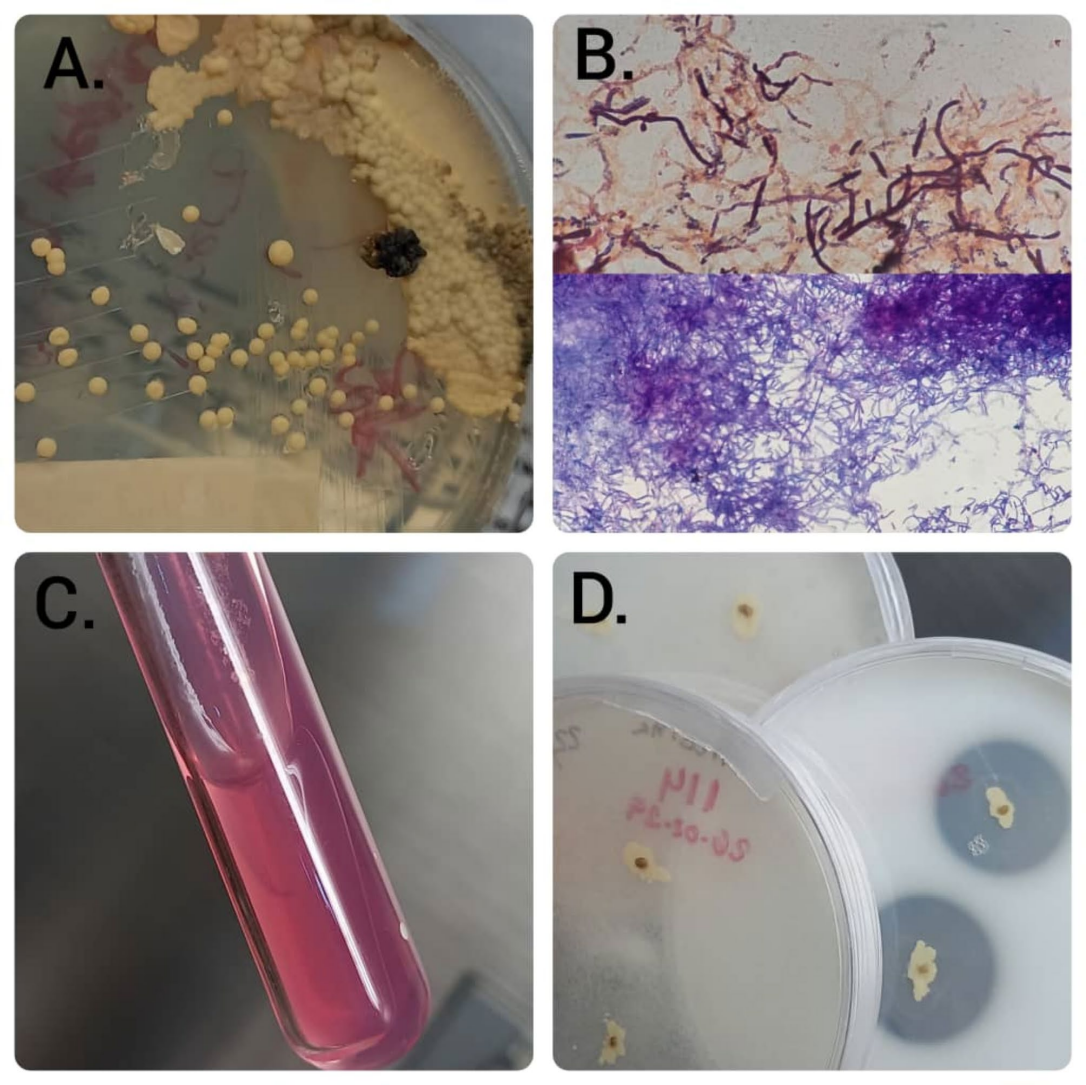
Phenotypic identification of *N. farcinica*. **(A)** Growth of slightly rough, dry, beige/brown colonies on Sabouraud agar. **(B)** Gram and Ziehl-Neelsen staining: microsiphoned gram-positive filaments, partially acid-fast. **(C)** Urea agar: positive. **(D)** Amino acids: casein: positive, tyrosine: negative, xanthine: negative

**Fig. 3:**
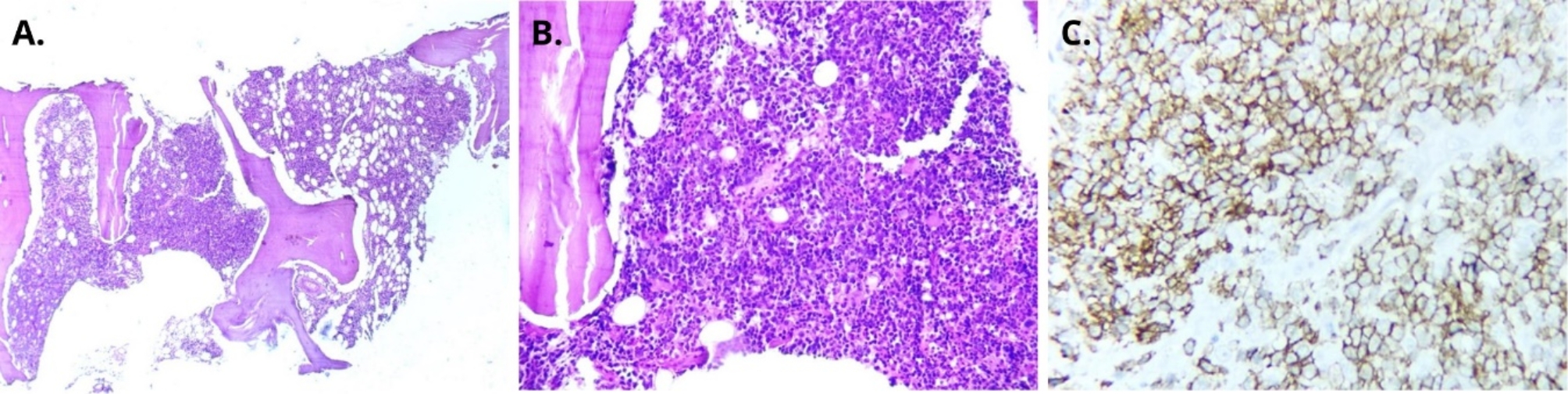
Histological study of bone marrow. **(A)** Presence of nodular aggregates with paratrabecular and interstitial localization, hematoxylin and eosin: 40X. **(B)** Medium to large cells with paratrabecular localization, hematoxylin and eosin: 400X. **(C)** Neoplastic cells expressing CD20 on the cytoplasmic membrane, 400X

Five days post-discharge, the patient experienced a recurrence of febrile episodes accompanied by increased abdominal girth and severe abdominal pain localized to the right hypochondrium. An abdominal ultrasound conducted on February 20, 2024, showed hepatomegaly, multiple retroperitoneal lymphadenopathies, and moderate ascites. A transthoracic echocardiogram revealed a moderate volume of free pericardial fluid. Subsequently, the patient developed altered consciousness characterized by somnolence and temporospatial disorientation, alongside progressive dyspnea, necessitating readmission on February 26, 2024. Paraclinical testing revealed leukopenia (white blood cell count: 2.3 × 10^3^/µL), anemia (hemoglobin: 8 g/dL), thrombocytopenia (platelets: 88 × 10^3^/µL), elevated creatinine (2.6 mg/dL), and a markedly elevated LDH (1,860 U/L). Despite supportive care, his clinical condition deteriorated, and he passed away on February 28, 2024, after 48 h of hospitalization. Postmortem, fungal culture report of a BM aspirate confirmed the isolation of *N. farcinica*. The BM sample was processed at the Department of Mycology of the Instituto Nacional de Higiene “Rafael Rangel” and cultured on Sabouraud agar with chloramphenicol, Mycosel agar, and brain heart infusion agar. After seven days of incubation at 25 °C, slightly rough, dry, beige-to-brown colonies were observed on Sabouraud agar (Fig. 2A). Microsiphonated filaments were identified upon direct examination. The colonies underwent Ziehl-Neelsen and Gram staining, revealing partially acid-fast and Gram-positive microsiphonated filaments (Fig. 2B). A urease test yielded positive results (Fig. 2C), providing presumptive identification of *Nocardia* spp. Further phenotypic identification using amino acid hydrolysis tests indicated the following results: casein hydrolysis positive, tyrosine and xanthine hydrolysis negative. Based on these conventional tests, the isolated organism was identified as *N. farcinica* [22] (Fig. 2D). Unfortunately, antimicrobial susceptibility testing could not be performed. Additionally, a BM biopsy report revealed increased lymphoid activity, with clusters of small lymphocytes occupying the trabecular space. Immunohistochemical analysis confirmed a diagnosis of large cell non-Hodgkin’s lymphoma of B-cell lineage (Fig. 3).

## Discussion and conclusions

We report here a rare case of disseminated nocardiosis with the isolation of *N. farcinica* in BM culture from a severely immunocompromised patient with HIV and B-cell non-Hodgkin’s lymphoma. Historically, the frequency of *Nocardia* spp. infection in HIV patients has been reported to range from 0.3 to 1.8% [23]. Since the advent of highly effective ART, individuals with HIV no longer account for a large proportion of reported cases [5]. Consistent with our case, most HIV patients with nocardial infections are severely immunocompromised. In case series, the mean CD4^+^ count is between 20 and 40 cells/µL [4, 5, 24]. In settings with a high HIV prevalence, the incidence ranges from 3,400 to 16,300 cases per 100,000 population in patients with suspected pulmonary disease [25, 26].

The microbiological diagnosis of *Nocardia* spp. is challenging due to its complex identification process in routine laboratories and its capacity to mimic other diseases. In this case, disseminated histoplasmosis was initially suspected, given the patient’s pancytopenia and markedly elevated LDH. *Nocardia* most commonly causes localized pulmonary disease [27], with typical findings such as cavitations and nodular lesions [28], which may mimic pulmonary tuberculosis [7]. Although cavitations were not observed in our patient, nodular disease was documented alongside bullous lesions of unclear etiology, potentially linked to chronic lung disease associated with heavy smoking. In patients with chronic pulmonary conditions (e.g., chronic obstructive pulmonary disease, sarcoidosis), *Nocardia* spp. may mimic exacerbation of the underlying disease, potentially delaying diagnosis [29]. Moreover, *Nocardia* spp. may spread contiguously from the lungs to adjacent structures [7], causing complications such as empyema, mediastinitis, and superior vena cava syndrome [2]. Upon readmission, our patient presented with pericarditis and pericardial effusion—an infrequent manifestation of *Nocardia* spp. infection that has been previously described [30, 31]. These findings highlight the importance of maintaining a high suspicion for nocardiosis in HIV-positive patients with pulmonary or pericardial involvement and CD4^+^ T-cell counts < 50/µL [32]. Other differential diagnoses, such as multicentric Castleman’s disease, could also have been considered. Multicentric Castleman’s disease, a rare lymphoproliferative disorder more common in HIV patients, frequently presents with fever, pancytopenia, lymphadenopathy, and ascites [33, 34].

In our patient, BM culture revealed infection with *N. farcinica*, a species previously documented in patients with AIDS [35]. Current guidelines recommend TMP-SMX as part of first-line therapy for nocardiosis [36]. However, susceptibility patterns vary significantly across reports. In a retrospective review of 765 *Nocardia* spp. isolates submitted to the United State Centers for Disease Control and Prevention between 1995 and 2004, 42% exhibited resistance to TMP-SMX, and 61% showed resistance to SMX [37]. *N. farcinica* is uniformly susceptible to amikacin but resistant to other aminoglycosides and third-generation cephalosporins [38]. Despite receiving oral TMP-SMX doses effective against *Nocardia* spp. for pneumocystosis prophylaxis, our patient’s condition deteriorated. Intravenous TMP-SMX combined with amikacin is typically recommended for disseminated or severe nocardiosis [39], yet we cannot exclude the possibility that the *N. farcinica* strain in this case was resistant to TMP-SMX. Unfortunately, antimicrobial susceptibility testing of the isolate was not performed. In addition to the antimicrobial challenge, several factors may have contributed to the adverse outcome. Infection with *N. farcinica*, a higher Charlson comorbidity index (elevated in this patient due to AIDS and non-Hodgkin’s lymphoma), and advanced-stage infection rather than mere dissemination are known predictors of increased 1-year mortality in nocardiosis [40, 41].

Non-Hodgkin’s lymphoma is one of the most common neoplasms in HIV-positive patients, with an incidence over 100 times higher than in the general population. Large B-cell and Burkitt’s lymphoma are the most frequently observed subtypes [42]. While *Nocardia* spp. infections have been associated with various hematological malignancies and solid tumors, lymphomas remain the most commonly linked malignancy [3]. Previous reports of *Nocardia* spp. infections in B-cell non-Hodgkin’s lymphoma patients often involve individuals receiving chemotherapy, particularly rituximab and ibrutinib, which are associated with increased susceptibility to *Nocardia* spp. [43–49]. However, cases linking *N. farcinica* to hematological malignancies remain limited to isolated case reports [50]. In this patient, the coexistence of B-cell non-Hodgkin’s lymphoma and HIV infection in a stage of severe immunosuppression likely synergistically contributed to the development of disseminated nocardiosis.

Several factors in our case could have contributed to the high short-term mortality observed. Infection with *N. farcinica*, a higher Charlson comorbidity index, and advanced-stage infection are well-established predictors of increased 1-year mortality following *Nocardia* spp. infection [40, 41]. In cases of non-Hodgkin’s lymphoma, prognosis is influenced by tumor-related factors such as subtype and stage [51]. The 5-year survival rate for non-Hodgkin’s lymphoma is significantly lower in people living with HIV compared to HIV-negative individuals (25.8 months vs. 75.4 months). Additionally, a higher International Prognostic Index, elevated LDH levels, BM involvement, an absolute lymphocyte count of < 1,000 cells/mm³, and B symptoms are all associated with shorter survival times [52, 53]. While survival in people living with HIV improves with ART [54], the clinical course in this case appeared to worsen after its initiation. This deterioration may be attributed to immune reconstitution inflammatory syndrome (IRIS), a condition that typically arises days to months after ART initiation. It is characterized by a gradual restoration of pathogen-specific immunity, where the immune system recognizes previously occult antigens. In our patient, it is plausible that disseminated nocardiosis and B-cell lymphoma were exacerbated by ART initiation, as has been previously documented [55]. In a large cohort of HIV-associated lymphoma patients, 12% met the criteria for IRIS-associated unmasking lymphoma. Although 5-year overall survival was similar between IRIS (49%) and non-IRIS cases (44%), early mortality was notably higher among IRIS cases [56]. However, in our case, the lack of HIV viral load measurements before and after treatment, as well as the absence of CD4^+^ T-cell monitoring post-ART, limits confirmation of this hypothesis. The risk of IRIS is strongly associated with CD4^+^ T-cell count at ART initiation, particularly at levels below 50 cells/µL [57]. In our patient, the low CD4^+^ count likely increased the risk of IRIS. These findings underscore the importance of initiating ART before advanced immunodeficiency develops to reduce the likelihood of IRIS and its associated complications.

Most studies on *Nocardia* species in Venezuela focus on the analysis of mycetoma samples [17, 18], a chronic granulomatous subcutaneous disease endemic to tropical and subtropical regions, including Venezuela. The disease is particularly prevalent in the central-western regions of the country, specifically in Lara and Falcon states. Additional cases have been reported in the Central, Zuliana, and Guayana regions [19]. Only one study has included samples of pulmonary nocardiosis, where 16 S ribosomal RNA analysis reclassified 53 strains from clinical cases, identifying *N. brasiliensis*, *N. otitidiscaviarum*, *N. farcinica*, and *N. nova* [20]. Due to low clinical suspicion of nocardiosis among physicians and limitations in molecular diagnostics for actinomycetes, the clinical and epidemiological behavior of the disease remains poorly understood in Venezuela. Strengthening diagnostic capabilities for *Nocardia* spp. infections in resource-limited settings, such as Venezuela, are critical for the timely implementation of targeted treatments.

In conclusion, information on nocardiosis in Venezuela is scarce, and to the best of our knowledge, no cases have been reported in HIV patients. We present the case of a patient with severe immunosuppression due to HIV who died without a definitive diagnosis. Post-mortem BM analysis revealed the isolation of *N. farcinica* and the presence of B-cell Hodgkin’s lymphoma. This case highlights the importance of early and accurate diagnosis of nocardiosis, as misidentification may lead to inappropriate treatment protocols and adverse patient outcomes.

## Data Availability

All data and materials in this article are included in the manuscript.

## Abbreviations

ART: Antiretroviral therapy
IRIS: Immune reconstitution inflammatory syndrome
TMP-SMX: Trimethoprim/Sulfamethoxazole
AIDS: Acquired immunodeficiency syndrome
HIV: Human immunodeficiency virus
LDH: Lactate dehydrogenase
BM: Bone marrow
